# Intestine-Liver Axis On-Chip Reveals the Intestinal Protective Role on Hepatic Damage by Emulating Ethanol First-Pass Metabolism

**DOI:** 10.3389/fbioe.2020.00163

**Published:** 2020-03-17

**Authors:** Vincenza De Gregorio, Mariarosaria Telesco, Brunella Corrado, Valerio Rosiello, Francesco Urciuolo, Paolo A. Netti, Giorgia Imparato

**Affiliations:** ^1^Center for Advanced Biomaterials for HealthCare@CRIB, Istituto Italiano di Tecnologia, Naples, Italy; ^2^Interdisciplinary Research Centre on Biomaterials (CRIB), University of Naples Federico II, Naples, Italy; ^3^Department of Chemical, Materials and Industrial Production Engineering (DICMAPI) University of Naples Federico II, Naples, Italy

**Keywords:** first-pass metabolism of ethanol (Et-OH), intestine-liver-on-chip, bottom-up tissue engineering approach, endogenous ECM, 3D tissue

## Abstract

Intestine-Liver-on-chip systems can be useful to predict oral drug administration and first-pass metabolism *in vitro* in order to partly replace the animal model. While organ-on-chip technology can count on sophisticated micro-physiological devices, the engineered organs still remain artificial surrogates of the native counterparts. Here, we used a bottom-up tissue engineering strategy to build-up physiologically functional 3D Human Intestine Model (3D-HIM) as well as 3D Liver-microtissues (HepG2-μTPs) *in vitro* and designed a microfluidic Intestine-Liver-On-Chip (InLiver-OC) to emulate first-pass mechanism occurring *in vivo*. Our results highlight the ethanol-induced 3D-HIM hyper-permeability and stromal injury, the intestinal prevention on the liver injury, as well as the synergic contribution of the two 3D tissue models on the release of metabolic enzymes after high amount of ethanol administration.

## Introduction

Orally administered drugs and chemicals are primarily metabolized by the gastrointestinal (GI) tract and liver (de Sousa and Bernkop-Schnürch, 2014). The xenobiotics and their metabolites interact with the small intestine, causing damage, and a fraction of these is transported across the gut epithelium and absorbed into the bloodstream before reaching the liver by the portal vein (Maurice et al., 2013). Therefore, there is a need to understand how such compounds may affect function at the organ level. Traditionally, testing the toxic effects of chemicals or drugs has relied on large-scale animal studies (Council, 2007). In addition to being extremely expensive, the data obtained with animal models often cannot be extrapolated to humans (Esch et al., 2011). In this perspective, there is a need to design *in vitro* organotypic cultures to investigate toxicity. In the last few years, *in vitro* models of the GI tract (Huh et al., 2011; Bhatia and Ingber, 2014) and liver (Santaguida et al., 2006; Inamdar and Borenstein, 2011) have been developed individually, but single-organ systems lack the complex intercellular and inter-organ communication that occurs *in vivo* and therefore are unable to properly model the enterohepatic circulation and first-pass metabolism that have been shown to be critical for assessing drug and xenobiotics metabolism. For this reason, recently, multi-compartment microfluidic-based devices (organs-on-a-chip) have been proposed to increase safety and the efficacy of the drug development process. These platforms consist of microfluidic cell culture devices, fabricated by means of microfabrication methods, which contain continuously perfused chambers inhabited by living cells arranged to simulate tissue- and organ-level physiology. By recapitulating the multi-cellular architectures, tissue–tissue interfaces, physicochemical microenvironments, and vascular perfusion of the body, these devices exceed the static conventional 2D or 3D culture systems (Esch et al., 2012). In an attempt to recapitulate the integration of the GI tract and the liver mimicking the first-pass metabolism, several *in vitro* models have been established. Choe et al. have recently developed a microfluidic device that consists of 2D cell models seeded into two separate compartments for gut epithelial cells (Caco-2) and liver cells (HepG2), respectively, designed so that drugs go through a sequential absorption in the gut chamber and metabolic reaction in the liver chamber (Choe et al., 2017). In another work, 3D gut model and Hepg2 layer were integrated into microfluidic device to recapitulate the complex process of absorption and metabolism of digested lipids on *in vitro* model of hepatic steatosis (Lee and Sung, 2018). Another noteworthy model built up by Shuler's group is based on pumpless modular GI–liver model to co-culture human intestinal cells and 3D hepatic cells in order to replicate human GI–liver physiology (Chen et al., 2018). On the other hand, van Midwoud et al. developed a microfluidic system for the sequential perfusion of rat intestinal and liver slices for metabolism studies (van Midwoud et al., 2010). However, this approach cannot be easily translated to the use of human tissue slices, whose viability dramatically decreases over a few days of culture, making this tissue model infeasible for organ-on-chip purposes. Other studies are based on first-pass metabolism of orally administered drugs by using multi-compartment pharmacokinetic models. In particular, Mahler et al. developed a microfluidic biochip in which HT-29 goblet-like cells are included in the GI-tract compartment lining the Caco-2 cell layer with mucous granules, in order to demonstrate absorption, distribution, metabolism, and toxicity (ADME-Tox) of acetaminophen (APAP) (Mahler et al., 2009). Also, Prot et al. analyzed the metabolism of paracetamol through a microfluidic device, which comprises the Caco-2 cells cultivated on a conventional cell culture insert introduced in a bioreactor and the liver cells modeled by HepG2/C3a isolated from rat/human primary hepatocytes. Their experiments were also supported by a mathematical model to estimate intrinsic *in vitro* parameters and to predict *in vivo* processes (Prot et al., 2014). In a recent work, an integrated gut–liver microphysiological systems was used to elucidate the inter-tissue cross-talk under inflammatory conditions, focusing on the long-term co-culture into the device to reproduce pathological conditions (Chen et al., 2017). However, even if these systems (Esch et al., 2014) provide important insights into the molecular mechanism of GI tract metabolism, they include over-simplified cell mono or co-culture models, thus failing in the replication of the 3D physiological ECM microenvironment with its dynamic complexity. Our studies have recently demonstrated the crucial role of the native ECM remodeling on the intestine adsorptive and metabolic function as well as the ECM involvement in the intestinal mucosal inflammation (De Gregorio et al., 2019). With the perspective of elucidating the inter-organs crosstalk with a more physiological functional 3D tissues, we reported a development of a novel Intestine-Liver-on-Chip (InLiver-OC) consisting of two directly interconnected chambers (Intestine-compartment and Liver-compartment) that enable the culture of a 3D Human Intestine Model (3D-HIM) as well as 3D liver microtissues (HepG2-μTPs) in order to simulate the physiological mechanism of the first-pass metabolism of orally-ingested compounds. Bottom-up approach was used to fabricate the 3D-HIM provided with an endogenous ECM (Imparato et al., 2013) and HepG2-μTPs, as previously reported (Corrado et al., 2019). The combination of such physiological relevant 3D models with microfluidic technology allowed us to investigate the protective role of the intestine on liver injury, analyzing the 3D-HIM hyper-permeability as well as the intestinal stroma remodeling. In addition, in this system, the integrated InLiver-OC was used to study the synergic contribution of the two 3D tissue models on the release of metabolic enzymes after high amount of ethanol (Et-OH) administration as exogenous stimulus mimicking the “binge drinking.” This multifunctional system has a broad applicability toward advancing fundamental understanding of human patho-physiology and drug development processes.

## Materials and Methods

### Cell Type

For 3D-HIM production, human intestinal myofibroblasts (H-InMyoFib) were purchased from Lonza and cultured in SMGMTM-2 BulletKitTM medium (Lonza). H-InMyoFibs at 3–5 passages were used. Human intestinal epithelial cells (Caco-2) were provided by American Type Culture Collection (ATCC) and were cultured in Dulbecco Modified Eagle Medium (DMEM, Microtech) with 10% of fetal bovine serum (FBS, Microtech), 100 μg mL^−1^ L-glutamine, 100 U mL^−1^ penicillin/streptomycin. For HepG2-μTPs production, hepatocellular carcinoma cells (HepG2) cells were purchased by ATCC and were cultured in Minimum Essential Medium Earle's Salt (MEM, Microtech), containing 10% fetal bovine serum, 100 μg mL^−1^ L-glutamine, 100 U mL^−1^ penicillin/streptomycin, 0.1 mM Non-Essential Amino Acid and 0.1 mM Sodium pyruvate. Cells were incubated at 37°C in a humidified atmosphere with 5% CO_2_.

### 3D Human Intestine Model (3D-HIM) Production

Gelatine porous microscaffolds (GPMs) (75–150 μm diameter) were made-up by a slightly adapted double emulsion method (O/W/O) and stabilized with glyceraldehyde at 4% as previously described (Imparato et al., 2013). Briefly, 10 mL of water containing TWEEN 85 (6% w/v) were used to dissolve the gelatin powder (type B, Mw 176,654 Da) (Sigma Aldrich) at 60°C. Then, an oil in water (O/W) emulsion was produced by adding a solution of Toluene and SPAN 85 (3% w/v) to the aqueous gelatin solution (8% w/v) (Sigma Aldrich). To obtain gelatin micro-beads, 30 mL of additional toluene were added (O/W/O) and cooled to 5°C. At last, to obtain the toluene extraction and gelatin micro-beads stabilization, ethanol (20 mL) was poured. Dried GPMs were sterilized with ethanol, washed with PBS, and then inoculated (2 mg/mL of GPMs; 5 × 10^3^ beads/mg) with H-InMyoFibs (1.5 × 10^5^ cell/mL) and cultured in a dynamic conditions in spinner flask bioreactor (CELLSPIN, 250 ml, Integra Biosciences) to obtain Human Intestine-μTPs (HI-μTPs) (De Gregorio et al., 2018a). In order to promote H-InMyoFibs adhesion, spinner flask operated with and intermittent stirring for 6 h post inoculation (5 min shaking, 40 min stationary) and continuous stirring at 20 rpm was set for 10 days. Spinner flask bioreactor operated in the cell incubator at 37°C and 5% CO_2_; the media replacement occurred every 2 days, and 2-O-alpha-D-Glucopyranosyl-L-ascorbic Acid 0.5 mM (TCI Europe) was added at each culture media change. After 10 days of spinner flask bioreactor culture, HI-μTPs were transferred into a maturation chamber bioreactor where HI-μTPs assembling took place forming disc-shaped (1 mm thick, 6 mm diameter) 3D Intestinal stroma (3D-ISs) (De Gregorio et al., 2018a). The maturation chamber was inserted into a spinner flask programmed at 60 rpm. After 2 weeks of culture, the maturation chamber was opened, the 3D-ISs were withdrawn and washed 2-folds with PBS solution, then transferred into a transwell insert (6.5 mm diameter; Corning), and left to dry for 5 min under laminar hood. Further, Caco-2 cells suspension (2 × 10^5^ cells in 50 μL) was seeded on each 3D-IS in order to produce the intestinal epithelium. The samples were incubated for about 2 h at 37°C, 5% CO_2_ to permit Caco-2 cells adhesion onto the 3D-IS apical surface. Further, submerged culture was performed by adding 200 μL of DMEM on the apical side of the 3D-IS and 600 μL of DMEM in the baso-lateral side for 7 days. Then, to induce the epithelial cells polarization and differentiation, the samples were cultured for 2 weeks in an air-liquid interface culture. Three times per week the culture medium was replaced. At the end of the experiment, the full-thickness intestinal equivalents (3D-HIMs) composed by 3D-IS overlapped by intestinal epithelium were taken from the transwell insert for further investigation or were loaded into the Intestine chamber of the Intestine-on-Chip (In-OC) or Intestine-Liver-on-Chip (InLiver-OC).

### HepG2-μTPs Development

HepG2 cells were cultured on GPMs in spinner flasks (Integra), as previously reported with slight modifications (Corrado et al., 2019). Briefly, 35 mg of GPMs were loaded with 5.25 × 10^6^ cells (30 cell/GPM ratio). To promote cell seeding on GPMs an intermittent stirring regime (30 min at 0 rpm, 5 min at 30 rpm) was applied for 24 h. Afterwards, the stirring speed was kept at a continuous 20 rpm for up to 7 days. Culture medium was changed three times per week. All cultures were maintained at 37°C in a humidified 5% CO_2_ incubator for 6–7 days, and then HepG2-μTPs were loaded into microfluidic Liver-On-Chip (Liver-OC) or into InLiver-OC.

### Microfluidic Device Fabrication

The microfluidic InLiver-OC device was fabricated by a rapid prototyping procedure. The PMMA master mold was designed by AutoCAD and carved with micromilling machine (Minithech CNC Mini-Mill) making a relief positive geometry. A mixture of PDMS pre-polymer and curing agent 10:1 (w/w) was prepared, degassed under vacuum for 20 min to remove air bubbles, and then poured on PMMA master. The set-up was incubated at 80°C for 60 min, then peeled off from master molds. The device consists of two compartments, the intestine compartment (Intestine_c_) and the liver compartment (Liver_c_), which are connected by a central microchannel. The Intestine_c_ was designed with a central microchannel (1.2 mm wide × 40 mm long × 0.6 mm high), which transported the medium into an intestine chamber (9.5 mm diameter × 5 mm high). The Liver_c_ communicated with the Intestine_c_ by a central microchannel, which was separated from three parallels chambers (0.5 mm wide × 0.6 mm high × 1 mm long) by micro-pillars (0.100 mm diameter × 0.09 mm pillar interspace). A collection channel (0.5 mm wide × 0.3 high) was used to collect tissue supernatants. Culture medium inlet and outlet, as well as inlet for HepG2-μTPs loading in the Liver_c_, were punched with a 2.5 mm biopsy punch (DifaCooper), while intestine chamber of the Intestine_c_ was punched using a 9.5-mm puncher (Am-Tech) (Figure S1). Then, a transwell insert was placed into the 9.5-mm holes of the biochip of the Intestine_c_. To ensure the selective transport of fluid through the 3D-HIM, a silicon gasket and a PMMA cylinder were fabricated. In detail, PDMS gasket was fabricated by punching 1-mm-thick PDMS layer, first with a 4-mm puncher (for the inner hole) and then with a 6.5-mm puncher (for the outer hole). PMMA hollow cylinder (5 mm high, 1 mm thick) was made up with micromilling machine. The PDMS biochip was sterilized by autoclave, while PDMS gasket and PMMA cylinder were sterilized by UV light. Before the experiments, InLiver-biochip was washed twice with PBS and then was filled with cell culture medium without encapsulating air bubbles. Then, the 3D-HIM was inserted in the transwell insert of the Intestine_c_, the PDMS gasket was placed on the surface of the 3D-HIM, and the PMMA cylinder was placed on the PDMS gasket. In addition, to avoid bacterial contaminations, the Intestine_c_ was closed by laying a 100-μm sterile PDMS sheet fabricated by spin coating of PDMS (750 rpm × 30 s) onto transwell insert of the Intestine_c_. The entire set-up was connected to a syringe pump that worked at flow rate of 5 μL/min, a reservoir was connected to the basal Intestine_c_, and another one was connected to the In-Liver outlet in order to collect the cell supernatants from each compartment. For each experiment, two parallel devices were used. To simulate oral ingestion and toxic damage to both Intestine_c_ and Liver_c_, Et-OH dissolved into 300 μL of culture medium (400 mM) was added on the apical side of the Intestine_c_. The InLiver-OCs were incubated for 24 h at 37°C in a humidified atmosphere containing 5% CO_2_. The control group InLiver-OC without Et-OH treatment was named “–Et-OH;” the 400-mM Et-OH treated sample was named “+Et-OH.” 3D-HIMs and HepG2-μTPs were collected and processed for immune-histotypical and molecular characterizations. In addition, the media collected were analyzed for metabolites produced on the apical side of the Intestine_c_, on the lower side of the Intestine_c_ as well as on the outlet of the InLiver-OC.

### Mathematical Model CFD Simulation

The commercial CFD COMSOL Multiphysics vers. Five was used to verify the experimental setup, the three-dimensional velocity and the oxygen gradients in the InLiver-OC. In the CDF analysis, the entire InLiver-OC bioreactor was divided into two different parts, a fluid domain, indicated by “f” (culture medium), and a tissue domain, indicated by “t” (tissue equivalents). The oxygen transport and the three-dimensional velocity were evaluated by combining different physics. Fluid flow in tissue-free regions was modeled by using steady state Navier–Stokes Equation (1); fluid flow in tissue domains was modeled by using transport in porous media Equation (2); oxygen transport was modeled by using Mass Transport-Convection/Diffusion application mode Equation (3); the generation term for oxygen was modeled by using Michelis-Menten kinetic Equation (4). Reference pressure was considered at device outlet (*p* = 0 Pa); no slip condition was adopted at the walls; up to the intestine chamber a fixed concentration of O_2_ was imposed to simulate an open chamber; equality for velocity and pressure was imposed at the Navier–Stokes (u, v, w, p)/Brinkman (u_2_, v_2_, w_2_, p_2_) interfaces. All values utilized in the simulation are reported in Table 1. Laminar flow with different flow rates was set at the inlet, and zero pressure was set at the outlet.

(1) $$\begin{array}{r} \mu_{f}\nabla^{2}\nu_{f}=\;\nabla P_{f} \end{array}$$

Where μ_*f*_ is the dynamic viscosity, ν_*f*_ is the fluid velocity, and *P*_*f*_ is the pressure. Brinkman Equation (2) was used to describe the flow through the porous medium:

(2) $$\begin{array}{r} \mu_{t}\nabla^{2}\nu_{t}-K_{t}\mu_{t}=\nabla P_{t} \end{array}$$

Where *K*_*t*_ is the hydraulic permeability, μ_*t*_ is the viscosity of the tissue, and *P*_*t*_ is the pressure.

**Table 1:** Mathematical modeling variables.

**3D HIM properties**	
Equilibrium O_2_ concentration [C_0_]	0.22 mol/m^3^
3D HIM Cell density [ρ]	0.2 × 10^14^ cell/m^3^
Diffusion coefficient (D_t_)	10^−10^ m^2^/s
Dynamic permeability [k_dv_]	0 m^2^
Porosity of the tissue [ε]	0.7
Effective viscosity in the fluid [μ_t_]	0.0016 Pa s
Effective hydraulic conductivity [k_t_]	10^−11^ m^2^
Maximum oxygen consumption rate (V_max_)	10^−18^ mol/cells*s
O_2_ concentration at *V*_*max*_/2 (*K*_*m*_)	10^−3^ mol/m3
**HepG2-μTPs properties**	
Effective hydraulic conductivity [k_t_]	10^−11^ m^2^
Porosity of the tissue [ε]	0.7
Dynamic permeability [k_dv_]	0 m^2^
Diffusion coefficient (D_t_)	10^−10^ m^2^/s
Effective viscosity in the fluid [μ_t_]	0.0016 Pa s
HepG2-μTPs cell density [ρ]	2.4 × 10^13^ cell/m^3^
Maximum oxygen consumption rate (Vmax)	2.77 × 10^−17^ mol/cells*s
O_2_ concentration at *V*_*max*_/2 (*K*_*m*_)	6.3 × 10^−3^ mol/m^3^
**Culture media properties**	
Oxygen diffusivity [D_f_]	10–9 m^2^/s
Dynamic viscosity [μ_f_]	0.001 Pa s

The oxygen concentration within the system was calculated using the following mass balance Equation (3):

(3) $$\begin{array}{r} \text{D}\nabla^{2}\text{C}-\nabla \left(\text{C}\nu \right)=-R \end{array}$$

Where *C* is the oxygen concentration, ν is the fluid velocity field that was set equal to ν*f* in the domain “f” and ν_t_ in the domain “t,” respectively. *D* is the diffusion coefficient of the oxygen, set as *D*_*f*_ in the domain “f” and D_t_ in the domain “t,” respectively. *R* is the volumetric oxygen consumption rate expressed by the Michaelis–Menten law, according to the following Equation (4):

(4) $$\begin{array}{r} R=\frac{\rho V_{max}C}{K_{m}+C} \end{array}$$

where *V*_*max*_ is the maximum oxygen consumption rate, and *K*_*m*_ is the concentration at which the oxygen consumption rate is half of *V*_*max*_, ρ is the cell density in the perfusion chamber. For each tissue, corresponding values were considered. The HepG2 setting values were established from Weise et al. (2013). R was set to 0 only in the fluid domain, since cells are present only in the tissue domain.

### Culture Media Selection for Co-culture in InLiver-OC

Two culture media (DMEM and EMEM) and different supplements were used for 3D-HIMs and HepG2-μTPs, respectively. In order to determine the optimal medium composition to flow into InLiver-OC, different media were tested such as: In-medium (Enriched_DMEM), Liver-medium (Enriched_EMEM), and their combination at the following ratio 1:1, 1:2, and 2:1 ratio (v/v). MTT and LDH assays were used to establish the medium condition that guarantees the highest cell viability and lowest toxicity.

### Et-OH Curve and Cell Viability Assessment on 2D vs. 3D Liver Models

In order to evaluate the cell viability after Et-OH treatment in 2D as well as in HepG2-μTPs culture, 3-(4,5-dimethylthiazol-2-yl)-2,5-diphenyltetrazolium bromide (MTT) and Lactate dehydrogenase (LDH) assays were used according to the manufacturer's instructions (Dojindo Molecular Technologies Inc., Rockville, MD). For MTT assay 2D cell cultures (HepG2 cells) were seeded at 5 x 10^3^ cells for each well in 96-well cell culture plate and cultured for 3 days prior to the start of the experiments. Based on the generation time of the HepG2, the total number of the cells at the beginning of the MTT test was ~ 2.52 × 10^4^. For 3D culture, HepG2-μTPs (~15 microtissues, 2.32 × 10^4^ total cells) were collected from the spinner flask at culture day 6 and loaded in 96-well cell culture plate. All samples were starved in culture medium with 0.2% FBS overnight and then, the medium with different Et-OH concentrations (0, 100, 200, 300, 400, 500, and 600 mM) was administered to 2D HepG2 and 3D HepG2-μTPs and were cultured for 24 h. For MTT assay, the optical density of each well sample was measured with a microplate spectrophotometer reader at 550 nm. Then, to evaluate the LDH release, the culture supernatants from 2D and 3D HepG2-Microtissues LDH activity was performed, using LDH Detection Kit (Sigma-Aldrich) according to the manufacturer's protocol. Briefly, 50 μL of sample or NADH standard were added to a 96-well. Then, reaction mix was added to each well, and after 2–5 min, the absorbance at 450 nm was measured using a microplate reader; in this way a *T*_*initial*_ was fixed. Subsequent measurements were performed once every 5 min. The final measurement [(A450) final] was the penultimate reading or the value before the most active sample was near or exceeds the end of the standard curve. The time of the penultimate reading was *T*_*final*_. The LDH activity was evaluated by the following Equation (5):

(5) $$\begin{array}{r} LDH=\frac{B\times Sample\;diluition\;factor}{Reaction\;time}\times V \end{array}$$

Where B is the amount (nmole) of NADH generated between *T*_*initial*_ and *T*_*final*_, reaction time is the difference between *T*_*final*_ and *T*_*initial*_, and *V* is the sample volume. ADH was expressed as mUnit/mL.

### Cytotoxicity Assessment of Intestine-Metabolized Et-OH on HepG2-μTPs

An indirect estimation of the undigested Et-OH that crosses the 3D-HIM, and of the Et-OH metabolites produced by EtOH-treated, 3D-HIM were determined by collecting supernatants from apical (3D-HIM_apical supernatants_) and basal side (3D-HIM_basal supernatants_) of 3D-HIM treated or not with 400 mM Et-OH at T_0_ or at 24 h. MTT was performed on HepG2-μTPs treated with the 3D-HIM supernatants. In addition, zone of Inhibition Test on *L. rhamnosus* were performed after the treatment of the bacteria with the 3D-HIM supernatants. For Zone of Inhibition assay, transparent zones of bacterial grown inhibition indicated the antibacterial activity of the Et-OH. Image J software was used for the measurement of the diameter of the inhibition zones.

### Cytotoxic and Immunohistotypical Analyses of 3D-HIM Cultured in InLiver-OC

3D-HIMs cultured in InLiver-OC were either treated or not treated with 400 mM Et-OH for 24 h, then supernatants were collected, and LDH was performed as reported in section Et-OH Curve and Cell Viability Assessment on 2D vs. 3D Liver Models. Moreover, 3D-HIMs (control groups and Et-OH-treated) were fixed in a solution of 10% neutral buffered formalin for 1 h, rinsed in PBS, dehydrated in an incremental series of alcohol (75, 85, 95, and 100% twice, each step 30 min at RT), then treated with xylene (30 min twice) and embedded in paraffin. Tissue sections of 5 μm thick were stained with Hematoxylin and Eosin (H&E) or Alcian blue, mounted with Histomount mounting solution (Bioptica) on coverslips, and the morphological features of 3D-HIMs were observed with a light microscope. To quantify the mucus secretion, samples were processed by using color deconvolution plugin (Image J®). Automatic thresholding was applied, and blue staining (indicating mucus secretion) was separated from other colors. Further, the mucus deposition was quantified as the mean percentage of blue-stained region. Ten sections were used, and at least five different fields were randomly examined in each section for each time point. For immunofluorescence assay, 3D-HIMs were withdrawn from the InLiver-OC and fixed with 4% paraformaldehyde for 20 min and then rinsed with PBS. The samples were then incubated with a permeabilization solution (0.2% Triton X-100 + 3% BSA + PBS) for 10 min. After blocked for 1 h at RT, primary antibody (Claudin-1, 1/40, Abcam Laminin V, 1/50, Abcam; MMP-9, 1/250, Abcam) was incubated for 1 h at RT. Then, secondary antibody incubation, donkey AlexaFluor 546-conjugated anti-rabbit IgG antibodies, for Claudin-1 and Laminin V and goat AlexaFluor 546-conjugated anti-mouse for MMP-9 were incubated for 1 h. Cells nuclei were detected by DRAQ5 staining (5 μm/mL, Sigma Aldrich).

#### Quantification of Tight Junction Formation

For epithelial barrier evaluation, the 3D-HIMs were withdrawn from the InLiver-OC, and the quality of tight junctions was assessed by means of Claudin-1 staining. Cell samples were immune-stained for Claudin-1 and examined with Leica (Confocal Leica TCS SP5 II femtosecond laser scanning system, Leica) confocal microscope. For each sample, the number of tight junction structures, defined as a completely enclosed ring of smooth, contiguous, apical Claudin-1 staining, were counted in three different fields under 20 X magnification (>50 cells per field), and the mean number of tight junction structures per field was determined. Experiments were performed three times, and the mean number of tight junction structures per field from three independent experiments was calculated.

#### Transepithelial Electrical Resistance

The transepithelial electrical resistance (TEER) of the 3D-HIM cultured in InLiver-OC was measured by using an Autolab PGSTAT101 (potentiostat/galvanostat, Metrohm) equipped with FRA32M module (frequency response analysis module), which allowed for obtaining values of electrical impedance on a proper range of frequencies (10 Hz−10 kHz). Measurements were carried out using the two electrodes setup in potentiostatic mode, with an amplitude of 0.3 V_RMS and an AC current range of 10 μA (to avoid damage of the 3D-HIM); two platinum electrodes (0.38 mm diameter and 99.9% purity) were used. In order to obtain TEER values, impedance curve (40 data, 10 points per decade) was fitted with non-linear least-square method using software Nova2.1. In particular, the adopted model consisted of a series of a resistor (resistance of blank solution), CPE element (interaction between solution and electrodes), and a parallel between a resistor (TEER) and a capacitor (cell membrane) (Figure S2).

#### ECM Microarchitecture of 3D-HIM

Two-photon microscopy (Leica TCS SP5 II coupled with a multiphoton microscope where the NIR femtosecond laser beam was derived from a tunable compact mode locked titanium: sapphire laser-Chamaleon Compact OPO-Vis, Coherent) was used to produce Second Harmonic Generation (SHG) images in which unstained collagen in 3D-HIMs (untreated or Et-OH-treated) were highlighted. We used an excitation wavelength of λ_ex_ = 840 nm (two photon) and collected the signal at emission wavelength in the range λ_em_ = 420 ± 5 nm. ImageJ was used for the quantification of the collagen fraction (CF) by measuring the collagen portion in the ECM space in a selected region of interest (ROI). The collagen portion in the ECM corresponds to bright pixels with respect to black pixels, which represent the non-collagen portion. The CF was expressed as the ratio of bright pixels to total pixels (bright pixels + black pixels) in terms of percent in the selected ROI. In addition, the collagen assembly degree (CAD) was evaluated by analyzing the intensity of the SHG signal. All SHG images were subjected to noise subtraction, and the average intensity was evaluated as described by Equation (6):

(6) $$\begin{array}{r} CAD\propto \;\bar{\text{I}}\;=\;\frac{\sum_{i=1}^{255}\left(I_{i}p_{i}\right)}{\sum_{i=1}^{255}\left(p_{i}\right)}\; \end{array}$$

where $\bar{\text{I}}$ is the average intensity, *I*_*i*_ is the intensity corresponding to the pixel, *p*_*i*_, while the index, *i* = x_i_, y_i_, runs in the gray value interval from 1 to 255. The intensity $\bar{\text{I}}$ of the collagen network is known to be proportional to the degree of assembly of the newly synthesized collagen (De Gregorio et al., 2019). To quantify the stroma related changes, we performed Gray-level-Co-occurrence Matrix (GLCM) texture analysis, by using the Image J plug-in “Texture” on SHG images. In this work, we calculated the correlation curve (COR) for distances ranging from 1 to 100 pixels in the horizontal (0°) and vertical (90°) direction of each optical section that cover a length of interest of 40 μm. In such spatial windows, the distance at which the correlation function falls off represents the correlation length of the texture. In particular, correlation curve was calculated vs. neighbor index, and correlation length was obtained by fitting data with an exponential law. The Equation (7) of COR is given, below:

(7) $$\begin{array}{r} COR=\;\underset{i,j}{\sum }\frac{\left(i-\mu_{i\;}\right)\left(j-\mu_{j}\right)p\left(i,j\right)}{\sigma_{i\;}\sigma_{j}} \end{array}$$

where μ_*i*_, μ_*j*_, σ_*i*_, and σ_*j*_ are given by Equations (3–6):

(8) $$\begin{array}{rcl} \mu_{i} & = & \sum i,ji*p\left(i,j\right) \end{array}$$

(9) $$\begin{array}{rcl} \mu_{j} & = & \sum i,jj*p\left(i,j\right) \end{array}$$

(10) $$\begin{array}{rcl} \sigma_{i} & = & \sqrt{\sum i,j{\left(1-\mu_{i}\right)}^{2}p\left(i,j\right)} \end{array}$$

(11) $$\begin{array}{rcl} \sigma_{j} & = & \sqrt{\sum i,j{\left(1-\mu_{j}\right)}^{2}p\left(i,j\right)} \end{array}$$

In particular *p*(*i, j*) is the probability of gray level *i* occurring next to gray level *j*, μ_*i*_, μ_*j*_, σ_*i*_, and σ_*j*_ are the means μ and standard deviations σ of column *i* and line *j* of the matrix, respectively. All parameters have a maximum value of 1 and a minimum value of 0 or −1. The COR curve is an index of the architecture of the network, with a fast decay for fine textures and slow decay for coarse structure. From the COR curve, it is possible to obtain the correlation length, λ, defined as the distance at which the COR decay is equal to 0.5.

### Morphological, Functional and Immunohistotypical Analyses on HepG2-μTPs Cultured in Liver-OC or InLiver-OC

HepG2-μTPs (control groups and Et-OH-treated) withdrawn from the Liver-OC or InLiver-OC were processed for histological and immunofluorescence analyses, as reported in section Cytotoxic and Immunohistotypical Analyses of 3D-HIM Cultured in InLiver-OC. For immunofluorescence assay, primary antibody (Claudin-1, 1/40, Abcam; P-gp, 1/50, Abcam) and secondary antibody (donkey AlexaFluor 546-conjugated anti-rabbit IgG antibodies for Claudin-1, goat Alexa Fluor 488-conjugated anti-mouse IgG antibodies for P-gp) were used. Cells nuclei were detected by DRAQ5 staining (5 μm/mL, Sigma Aldrich). Furthermore, in order to measure albumin and urea released by HepG2-μTPs, culture media were collected after Et-OH administration on HepG2-μTPs cultured in Liver-OC. Specifically, the supernatants were centrifuged at 2,000 g for 10 min in order to remove cell debris. They were then stored at −20°C before being analyzed. Albumin secretion in cell culture supernatants was quantified by a sandwich enzyme-linked immunosorbent assay (ELISA) kit (Abcam), according to the manufacturer's instructions. All experiments were performed in triplicate. Urea levels in cell supernatants were determined by Quanti Chrom TM Urea Assay Kit (DIUR-500) according to the manufacturer's instruction.

#### Oil Red Staining on HepG2-μTPs

In order to evaluate the lipids production and localization in HepG2-μTPs within InLiver- and Liver-OC treated or not treated with 400 mM Et-OH for 24 h, oil red staining was performed. A group of samples was stained directly into the device, and another group was fixed in formalin 10%. The paraffin embedded samples were cut into 4-μm-thick sections, which were mounted on coating slides, unmasked with antigen retrieval and stained with oil red (Sigma Aldrich). Briefly all the samples were incubated for 5 min with 60% isopropanol at room temperature, rinsed in dH_2_O, and stained for 30 min with oil red working solution. Then, the samples were counterstained with hematoxylin, washed thoroughly with dH_2_O, and transferred to mounting medium (Bio Optica).

#### Reactive Oxygen Species (ROS) Production on HepG2-μTPs

HepG2-μTPs were collected from InLiver- or Liver-OC with or without 400 mM Et-OH treatment for 24 h, in order to assess Reactive Oxygen Species (ROS) production. After the incubation, all samples were washed with PBS and incubated with 10 μM CM-DCFDA (5-6)-chloromethyl-2,7 dichlorodihydrofluoresceine diacetate (ROS indicator, Invitrogen) at 37°C for 60 min in dark condition. For positive control groups, the samples were incubated with H_2_O_2_ 400 μM for 30 min. Then, all samples were washed in PBS, returned in pre-warmed growth medium, and visualized under confocal microscope (Confocal Leica TCS SP5 II femtosecond laser scanning system, Leica), using excitation appropriate source for fluorescein (FITC). For quantitative analysis, 20 images were processed by using ImageJ software, and the green signal (ROS indicator excitation at 485 nm) was normalized to the surface area.

### Metabolic and Molecular Assay on 3D-HIMs and HepG2-μTPs Cultured in InLiver-OC

#### Alcohol Dehydrogenase Release Assay

In order to evaluate the alcohol dehydrogenase (ADH) release, the culture supernatants from In-Liver-OC treated or not treated with 400 mM Et-OH for 24 h were collected and stored at −20°C before being analyzed. ADH activity was performed, using Alcohol Dehydrogenase Activity Detection Kit (Abcam), according to the manufacturer's protocol. Briefly, 50 μL of sample or NADH standard were added to a 96-well. Then reaction mix was added to each well, and the absorbance at 450 nm was measured using a microplate reader after 2–5 min; in this way, a *T*_*initial*_ was fixed. Subsequent measurement was performed after 2 h. The ADH activity was evaluated by the following Equation (12):

(12) $$\begin{array}{r} ADH=\frac{B\;\times Sample\;diluition\;factor}{Reaction\;time}\;\times V \end{array}$$

where *B* is the amount (nmole) of NADH generated between *T*_*initial*_ and *T*_*final*_, reaction time is the difference between *T*_*final*_ and *T*_*initial*_, and *V* is the sample volume. ADH was expressed as mUnit/mL. All experiments were performed in triplicate.

#### Reverse Transcription-Polymerase Chain Reaction Analysis

FFPE tissues blocks (3D-HIMs and HepG2-μTPs) was deparaffinized, digested, and total RNA was extracted by using RNeasy FFPE kit (Qiagen, Valentia, CA). Agarose gel electrophoresis assessed RNA integrity, and RNA concentrations were determined by UV light BioRad Imaging System (Biorad). 0.2 μg of total cellular RNA were reverse-transcribed (Expand Reverse Transcriptase, Roche Diagnostics; Milan, Italy) into complementary DNA (cDNA) using random hexamer primers (Random hexamers, Roche Diagnostics; Milan, Italy), at 42°C for 45 min. Two microliter of cDNA was amplified in a final volume of 50 μl composed by 10 mM Tris-HCl (pH 8.3), 1.5 mM MgCl_2_, 50 mM KCl, 200 μm dNTP and 2.5 U of Taq DNA polymerase (Roche Diagnostics). The reaction was carried out in a DNA thermal cycler (Applied Biosystem). The expression of the following genes was examined: MRP, P-gp, and glyceraldehyde-3-phosphate dehydrogenase (GAPDH) used as a housekeeping gene. The primers used for amplification were: MRP, 5′- tgatgagccgtatgttttgc−3′ and 5′- cttcggaacggacttgacat−3′; P-gp, 5′- atatcagcagcccacatcat−3′ and 5′- gaagcactgggatgtccggt−3′; GAPDH, 5′- ccacccatggcaaattccatggca−3′, and 5′- tctagactggcaggtcaggtccacc−3′. PCR products were analyzed by electrophoresis on 1.8% agarose gel in TBE. Densitometric analysis of ethidium bromide-stained agarose gel was carried out using Image J analysis and normalized by the housekeeping gene GAPDH. Each data point represented the mean-standard error of the mean of three biological replicates.

#### Solid Biomass Evaluation

In order to determine the biomass weight of dehydrated samples, supernatants from In-OC, Liver-OC, or In-Liver-OC were collected and centrifuged at 13,000 rpm per 15 min. All pellets were dehydrated in an incremental series of alcohol [30, 50, 75, 85, 95, and 100% twice], each step 30 min at RT, and then were made to evaporate under a chemical hood for 24 h. After that, they were weighed on an analytical balance (Xs105 dual range, Mettler Toledo).

### Statistical Analyses

Results were expressed as the mean ± standard deviation (s.d.) from three or more independent experiments (*n* ≥ 3). For section staining, three specimens were used for each experiment and five sections were stained per specimen, then about five ROIs were examined for each section. All results were then statistically analyzed by the Student's *t*-test. The differences between two or more groups were evaluated using one-way analysis of variance (ANOVA) followed by the Tukey's post-test. Statistical significance was set at a value of *p* < 0.05.

## Results

### Assessment of Intestinal and Epithelial Markers in 3D-HIM and HepG2-μTPs

**Figure 1:**
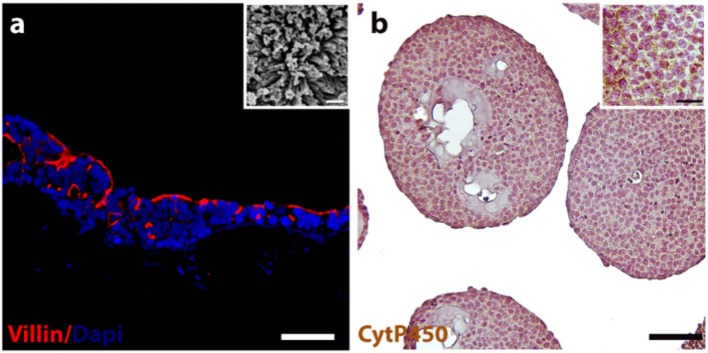
3D-HIM and HepG2-μTPs functionality. Immunofluorescence analysis reveals the epithelial cells marker stained in red (villin) and nuclei in blue (DAPI) **(a)**; scale bar, 75 μm. High magnification SEM micrograph shows the brush border on the apical side of the villi-like structures of the 3D-HIM (**a**, inset); scale bar 200 nm; representative immune-histochemical image of Cyt P450 on HepG2-μTPs **(b)** and high magnification inset, scale bar 75 and 50 μm, respectively.

Immunofluorescence staining was performed to verify the expression and localization of a representative intestinal epithelial marker, such as villin. As depicted in Figure 1a, the positivity for villin signal suggested the Caco-2 differentiation toward enterocytes cells showing highly polarized epithelial cells. In addition, the presence of microvilli structure on the free surface of the epithelial cells was also evidenced by ultrastructural SEM analysis, as showed in Figure 1a inset, confirming the epithelial polarization. The morphological features of the HepG2-μTPs was investigated via immune-histochemical analysis. A detectable expression of the Cyt P450, a membrane-associated protein correlated with toxic compounds metabolism, was displayed (Figure 1b, ^*^high magnification inset).

### InLiver-OC Assembly, Operation, and Comsol Simulation

**Figure 2:**
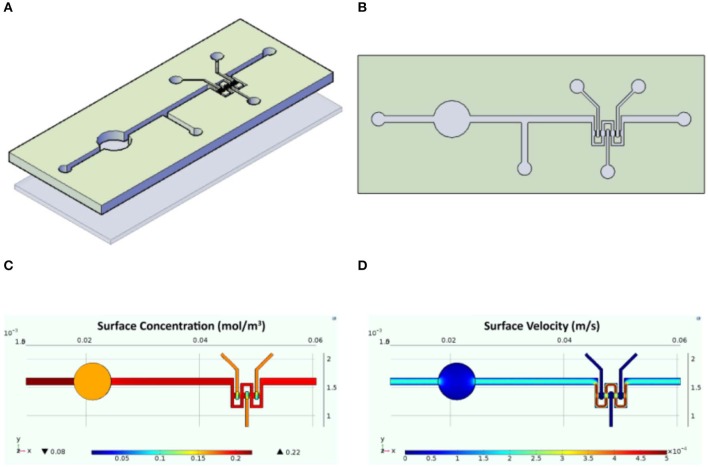
Schematic representation of the InLiver-OC and CFD study. Lateral **(A)** and top **(B)** view of the InLiver biochip with Intestine_c_ (In_c_) and Liver_c_ (Liv_c_); fluid dynamic simulation indicates the velocity field in the device system at a flow rate of 5 μL/min **(C)** and the oxygen concentration field in the device working at a flow rate of 5 μL/min **(D)**.

The designed InLiver-OC device allowed the co-culture of the two 3D organ models (3D-HIM in the Intestine_c_ and HepG2-μTPs in the Liver_c_) into separate compartments connected by means of microfluidic channel allowing an efficient unidirectional transport from the 3D-HIM to the HepG2-μTPs, mimicking the first-pass metabolism (Figures 2A,B). The Intestine_c_ presents a central hole to accommodate a commercially available transwell insert in order to obtain two separate Intestine side (apical and basal). The Liver_c_ was located downstream to the Intestine_c_ and was connected through a microchannel to the basal side of the Intestine_c_. 3D-HIM and HepG2-μTPs were produced as previously reported (De Gregorio et al., 2018a; Corrado et al., 2019), and when the best performance in terms of viability and functionality (5 weeks for 3D-HIM and 5–7 days for HepG2-μTPs) were detected, 3D-HIM was aseptically transferred into the apical side of the Intestine_c_, and HepG2-μTPs were loaded in each chamber of the Liver_c_ by pipetting. In order to assure the optimal conditions for cell survival, CFD (Figure 2) was used for simulating fluid dynamic conditions and oxygen concentration profile inside the InLiver-OC device. The flow rate at inlet (5 μL/min) assured the oxygen supply to both 3D models. As showed in Figure 2C, the O_2_ concentration in the 3D-HIM was 0.16 mol/m^3^, while the minimum O_2_ concentration in the HepG2-μTPs was 0.12 mol/m^3^. In Figure 2D, the velocity profile was shown, the maximum value of the fluid flow along the central channel was 5.4 × 10^−4^ m/s which is safe for cell viability according to literature (Sibilio et al., 2019).

### Selection of Medium Mixing for the Optimal 3D Co-culture in InLiver-OC

**Figure 3:**
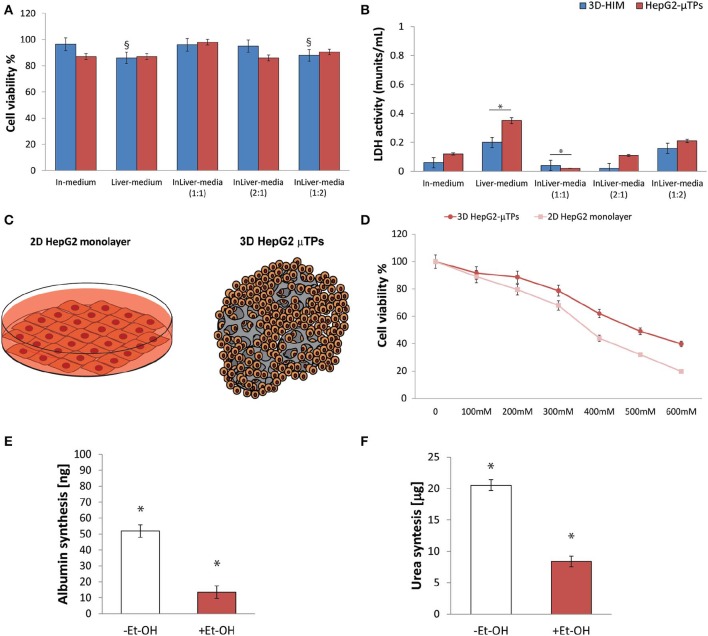
Selection of optimal media combination in InLiver-OC, HepG-2 cytotoxicity under different Et-OH concentrations and 3D HepG2-μTPs metabolic activity under 400 mM Et-OH. MTT cell viability **(A)** and LDH **(B)** assays on 3D-HIM and HepG2-μTPs cultured with different media combinations (^§^*p* < 0.05; *p < 0.05); schematic representation of standard culture dishes (2D HepG2 monolayer) and 3D configuration (3D HepG2-μTPs) **(C)**; dose response on 2D HepG2 monolayer vs. 3D HepG2-μTPs at different Et-OH concentrations (0–600 mM) **(D)**; comparison of Albumin **(E)** and urea production **(F)** on untreated (–Et-OH) and 400 mM Et-OH-treated (+Et-OH) 3D HepG2-μTPs. The Albumin amount is expressed in “ng” and the urea synthesis is indicated in “μg.” All the experiments were performed in triplicate (*n* = 3), Values represent the mean and the standard deviation (*p < 0.05).

Preliminary experiments were carried out in order to select the optimal media combination that did not cause the metabolic waste accumulation into the Liver_c_. MTT and LDH assays (Figures 3A,B, respectively) were performed to establish the best medium combinations. We found that when 3D-HIM was cultured with the Liver-medium (Enriched_EMEM) or with InLiver-media (Enriched_DMEM:Enriched_EMEM at ratio 1:2), the viability of 3D-HIM was marginally reduced (86 ± 7.12% and 88 ± 6.22%, respectively), and low value of LDH was detected (0.2 ± 0.04 and 0.16 ± 0.02 munits/mL, respectively). At the same media combination, the HepG2-μTPs viability was slightly affected (87 ± 4.44% and 90.5 ± 4.2%, respectively), and a small but significant amount of LDH (0.35 ± 0.18 and 0.21 ± 0.07 munits/mL, respectively) was detected, indicating that the 3D-HIM probably produced a small amount of metabolic waste that slightly induced the epithelial cell injury of the HepG2-μTPs. In addition, when HepG2-μTPs were cultured with In-medium (Enriched_DMEM) or with InLiver-media (Enriched_DMEM:Enriched_EMEM at ratio 2:1), a slight amount of LDH was revealed (0.12 ± 0.02 and 0.11 ± 0.04 munits/mL, respectively), although these values were not indicative of cellular damage. Therefore, the optimal media combination resulted in the InLiver-media (Enriched_DMEM:Enriched_EMEM at ratio 1:1) that did not affect viability (96 ± 8.3% for 3D-HIM, 98 ± 6.5% for HepG2-μTPs) and did not lead to the production of LDH (0.04 ± 0.01 for 3D-HIM and 0.02 ± 0.005 munits/mL for HepG2-μTPs, respectively) as reported in Figures 3A,B, respectively.

### Selection of the Optimal Et-OH Concentration for Evaluating HepG2-μTPs Damage

Initial experiments were focused on understanding how the HepG-2 cells respond to different concentration of Et-OH. In this experimental phase, the cytotoxic effect of Et-OH was assessed on standard culture dishes (2D HepG-2 monolayer) or in 3D configuration (3D HepG2-μTPs) (Figure 3C) by MTT viability assay. In detail, the 2D HepG-2 monolayer were seeded in 96 multiwell plate for 60 h in order to obtain 2.5 × 10^4^ cells for each well. Instead, the 3D HepG2-μTPs were cultured in a spinner flask in dynamic conditions in order to obtain 1,550 cell/μTPs; then on the sixth day, 5 HepG2-μTPs were added for each liver chamber for a total of 15 HepG2-μTPs (2.32 × 10^4^ total number of cells), as shown in the Figure S3. For 2D HepG-2 monolayer, a dose-dependent reduction of cell viability was reported from 0 to 600 mM Et-OH. For 3D HepG2-μTPs, a slight reduction of viability was revealed by adding 200 mM Et-OH, but an increase in cell cytotoxicity was shown at concentration over 300 mM. Specifically, when 2D HepG-2 monolayer was treated with Et-OH 400 mM, a significant reduction in cell vitality (44 ± 4.08%) compared to 3D HepG2-μTPs treated with the same concentration (61.9 ± 5.02%) was revealed. In the groups treated with 500 mM Et-OH, the viability percentage of the cells decreased at 32 ± 4.3% in 2D HepG-2 monolayer and at 49.07 ± 5.32% in 3D HepG2-μTPs. Finally, the cells treated with 600 mM showed a mortality that exceeds 80% in 2D HepG-2 monolayer and was around 60% in 3D HepG2-μTPs. In order to perform the experiment in conditions that guarantee the cells functionality by preserving more than 50% of cell viability, the Et-OH concentration of 400 mM was selected.

### 400 mM Et-OH Effect on HepG2-μTPs Metabolic Activity in Liver-OC

In order to determine the liver functionality, the response of the HepG2-μTPs to Et-OH treatment at 24 h was analyzed by measuring albumin and urea production. As shown in Figures 3E,F, the untreated samples (–Et-OH) exhibited high levels of albumin (51.84 ± 3.9 ng) and urea (20.52 ± 2.3 μg). In contrast, there was a significant decrease in the metabolic activity in 400 mM Et-OH-treated samples (+Et-OH). In particular, albumin production decreased at value of 13.56 ± 3.81 ng and urea at 8.39 ± 1.48 μg, demonstrating that the HepG2-μTPs responded to the toxic stimulus by reducing their metabolic activity.

### Intestine-Metabolized Et-OH Affects HepG2-μTPs Viability and *L. rhamnosus* Growth

**Figure 4:**
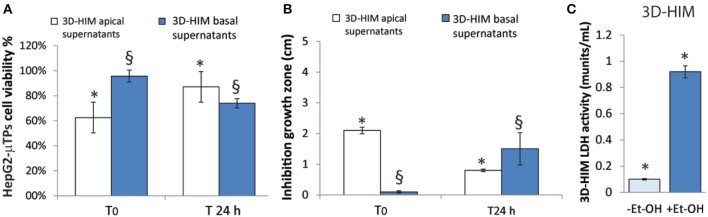
3D-HIM-metabolized Et-OH effect on HepG2-μTPs cytotoxicity and *L. rhamnosus* growth and Et-OH damage on 3D-HIM. MTT cell viability assay displays indirect estimation of the indigested Et-OH treating HepG2-μTPs with 3D-HIM supernatants (3D-HIM_apical_ and -_basal supernatants_) at two different time points (T_0_ and 24 h) (*p < 0.001; ^§^*p* < 0.05) **(A)**; Zone of Inhibition Test shows the antimicrobial activity of the indigested Et-OH treating HepG2-μTPs with 3D-HIM supernatants (3D-HIM_apical_ and -_basal supernatants_) on *L. rhamnosus* growth at two different time points (T_0_ and 24 h) (*p < 0.001; ^§^*p* < 0.05) **(B)**; LDH assay indicates the 3D-HIM toxicity after 400 mM Et-OH treatment **(C)** (*p < 0.01).

An indirect estimation of the undigested Et-OH crossing the 3D-HIM was evaluated by treating HepG2-μTPs and *L. rhanmosus* with tissue supernatants in order to perform cell viability assay (MTT) and Zone of Inhibition Test, respectively (Figures 4A,B). Specifically, HepG2-μTPs were treated with the 3D-HIM supernatants collected from apical (3D-HIM_apical supernatants_) or basal side (3D-HIM_basal supernatants_) of the Intestine_c_ at T_0_ or after 24 h of 400 mM Et-OH treatment. The results indicated that at T_0_ the 3D-HIM_apical supernatants_ slightly reduced HepG2-μTPs viability, resulting in cell vitality of 62.5 ± 5.2%. This value corresponded in the Et-OH dose response curve to an Et-OH concentration of 400 mM (Figure S4). No significant reduction in cell viability was induced by treating HepG2-μTPs with 3D-HIM_basal supernatants_ harvested at T_0_. HepG2-μTPs treated with 3D-HIM_apical supernatants_ collected at 24 h, revealed a very slight reduction of cell viability (87 ± 5.4% cell viability), while HepG2-μTPs treated with 3D-HIM_basal supernatants_ harvested after 24 h of Et-OH treatment, presented a more pronounced reduction in cell viability (74 ± 5.3% cell viability). The latter value corresponded in the dose response curve (Figure 3D) to an Et-OH concentration of 350 mM, suggesting that a small amount of Et-OH was metabolized by the 3D-HIM (Figure 4A) at 24 h. Further, the antibacterial action of Et-OH was evaluated by analyzing the supernatants collected from 3D-HIM_apical_ treated or not treated with 400 mM Et-OH at T_0_ and 24 h. The maximum zone of *L. rhamnosus* strain inhibition growth (2.1 ± 0.51 cm) was found by using supernatants collected at T_0_ from Et-OH treated 3D-HIM_apical_ while a relatively lower (0.8 ± 0.66 cm) growth inhibition zone was found by using supernatants collected from Et-OH treated 3D-HIM_apical_ at 24 h. Moreover, there was no antibacterial effect of the 3D-HIM_basal supernatants_ at T_0_, but an inhibition of the growth zone (1.5 ± 0.5 cm) was observed when 3D-HIM_basal supernatants_ at 24 h was used. Taken together, these results demonstrate that Et-OH or Et-OH products such as acetaldehyde reduce the growth of *L. rhamnosus* strain (Figure 4B).

### Et-OH-Induced 3D-HIM Hyper-Permeability and Stromal Injury in InLiver-OC

**Figure 5:**
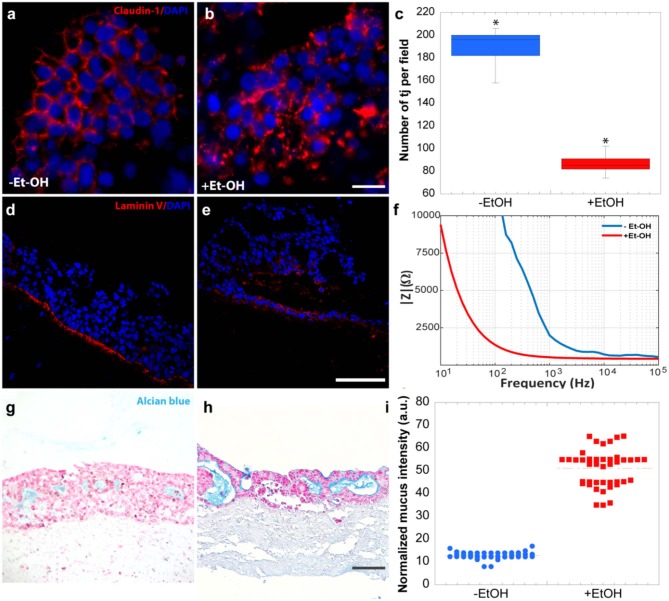
Et-OH-induced 3D-HIM hyper-permeability at epithelial level. Comparison of the expression of the intestinal epithelial tight junction cell marker (Claudin-1) on untreated (–EtOH) 3D-HIMs **(a)** and 400 mM Et-OH-treated (+Et-OH) 3D-HIMs **(b)**; scale bar 50 μm. Quantitative analysis of tight junction per field (*p < 0.05) **(c)**; confocal microscope analysis of immunofluorescence staining shows the Laminin V signal on untreated (–EtOH) 3D-HIMs **(d)** and 400 mM Et-OH treated (+Et-OH) 3D-HIMs **(e)**; cell nuclei stained with DAPI scale bar 75 μm; Quantitative analysis of TEER values **(f)**; Alcian blue-stained cross-sections reveals the mucus deposition in untreated (–EtOH) 3D-HIMs **(g)** and 400 mM Et-OH-treated (+Et-OH) 3D-HIMs **(h)**; scale bar 100 μm; Quantitative analysis of Alcian blue-stained samples reveals the mucus production **(i)**. All the experiments were performed in triplicate (*n* = 3); values represent the mean and the standard deviation.

**Figure 6:**
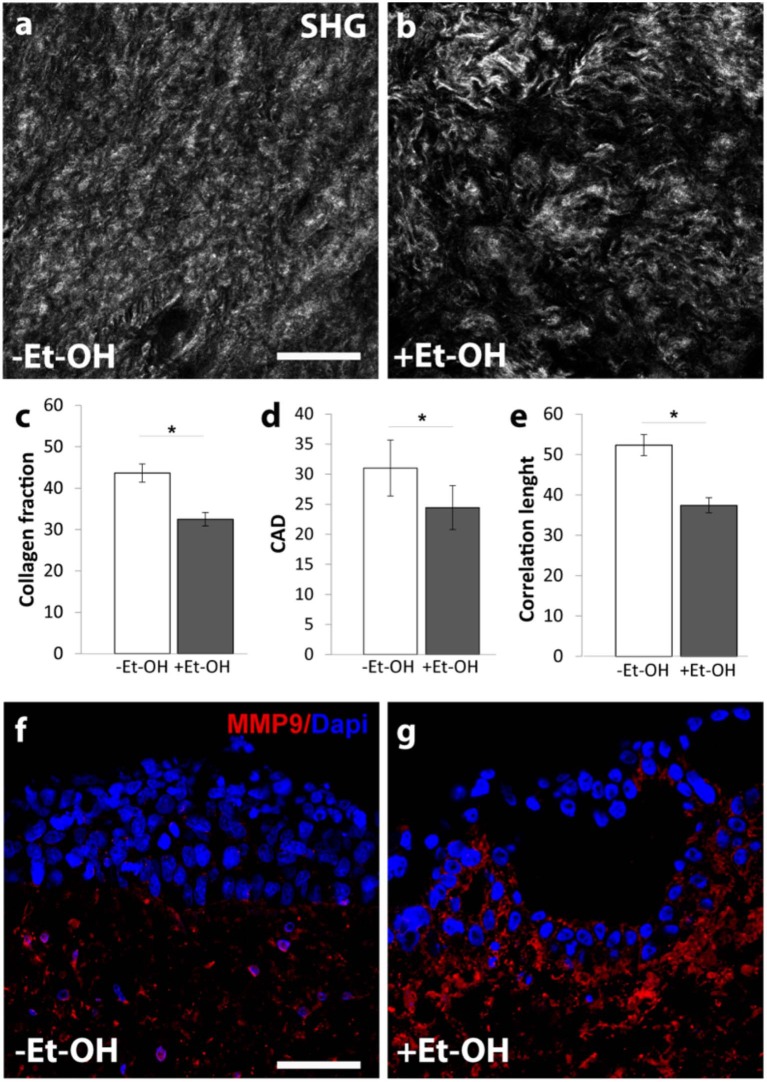
Et-OH-induced 3D-HIM stromal injury. Representative high magnification SHG images (gray scale) of the auto-produced collagen in untreated (–EtOH) 3D-HIMs **(a)** and 400 mM Et-OH-treated (+Et-OH) 3D-HIMs **(b)**; scale bar 75 μm; **(c)** Collagen fraction, **(d)** CAD and **(e)** GLCM analysis in untreated (–EtOH) and 400 mM Et-OH-treated (+Et-OH) 3D-HIMs (*p < 0.001); immunofluorescence images indicate different protein expression stained in red (MMP9) on untreated (–EtOH) 3D-HIMs **(f)** and 400 mM Et-OH-treated (+Et-OH) 3D-HIMs **(g)**; scale bar is 75 μm; DAPI (blue fluorescence). All the experiments were performed in triplicate (*n* = 3); values represent the mean and the standard deviation.

In order to evaluate the harmful effect of 400 mM Et-OH on 3D-HIM, the cell damage was assessed by LDH assay. The Et-OH was given on the apical side of the 3D-HIM, and at this dose, an LDH release of 0.92 ± 0.07 mU/mL at 24 h was found (Figure 4C), suggesting slight Et-OH-induced cell damage. This Et-OH concentration also impacted 3D-HIM's morphology and functional performance. In order to evaluate the effect in terms of the intestinal barrier integrity, intestinal tight junctions were investigated. High intensity of Claudin-1 signal, a tight junction marker, was observed on untreated samples (–Et-OH) compared to the Et-OH-treated ones (+Et-OH), in which the higher amount of acetaldehyde compromises the tight junction between the Caco-2 cells, determining an increase in barrier permeability (Figures 5a,b, respectively). Quantitative immunofluorescence analysis showed that the number of the tight junctions found on the untreated samples (189 ± 14.8, number of tight junctions per field) was higher compared to Et-OH-treated 3D-HIM (82 ± 8.01, number of tight junctions per field) (Figure 5c). Further, immunofluorescence staining for the Laminin V along the interface between stroma and epithelium indicated the presence of an intact basement membrane with a polarized epithelium on the untreated sample (-Et-OH, Figure 5d). In contrast, Et-OH treatment compromised the basement membrane integrity, as shown by pixelated signal of Laminin V and revealed the separation of the supra-basal epithelium from the basal lamina, which ultimately could cause the epithelium rupture (Figure 5e). In addition, an altered epithelial organization was also detected in Et-OH-treated 3D-HIM. TEER measurements were performed in order to confirm the damage at epithelium permeability. In agreement with immunofluorescence results, the untreated 3D-HIM (–Et-OH) expressed higher TEER values (4.72 kΩ) compared to Et-OH-treated 3D-HIM (43.4Ω) (Figure 5f), confirming that Et-OH treatment affected paracellular junctions. On the contrary, the capacitance measurements on untreated and treated samples resulted comparable (1.1 and 1.34 μF, respectively), suggesting that the membranes of the epithelial cells remain intact. Furthermore, in order to evaluate the mucus secretion on untreated (–Et-OH) and Et-OH-treated samples (+Et-OH), Alcian blue staining was performed. 3D-HIM respond to Et-OH treatment by overproducing mucus on Et-OH-treated 3D-HIM compared with the untreated one (Figures 5g,h, respectively). Quantification analysis of the Alcian blue images further confirmed the qualitatively observation of the histological images (Figure 5i). Moreover, analysis of the composition and architecture of the ECM was performed. Figure 6 reported the Second Harmonic Generation (SHG) images rising from newly formed collagen signal on the untreated (–Et-OH, Figure 6a) and Et-OH-treated 3D-HIM (+Et-OH, Figure 6b). The SHG images were exploited to estimate the CF, the CAD, and the correlation length of collagen network in the ECM (Figures 6c–e). Statistically significative differences were found between the values of untreated and Et-OH-treated samples. Both CF and CAD were affected by Et-OH treatment, 43.64 ± 6.6% vs. 32.48 ± 5.0%, the former, and 31.00 ± 7.6 vs. 24.43 ± 3.0 a.u, the latter. In addition, the correlation length highlights differences in collagen texture, reporting a value for untreated samples of 52.34 ± 2.50 a.u. and a value for Et-OH-treated samples of 37.40 ± 2.10 a.u (Figure 6e). Lastly, Et-OH treatment induced MMP-9 overexpression, as reported in Figures 6f,g.

### Protective Role of 3D-HIM on Et-OH-Induced Hepatic Cytotoxicity in InLiver-OC

**Figure 7:**
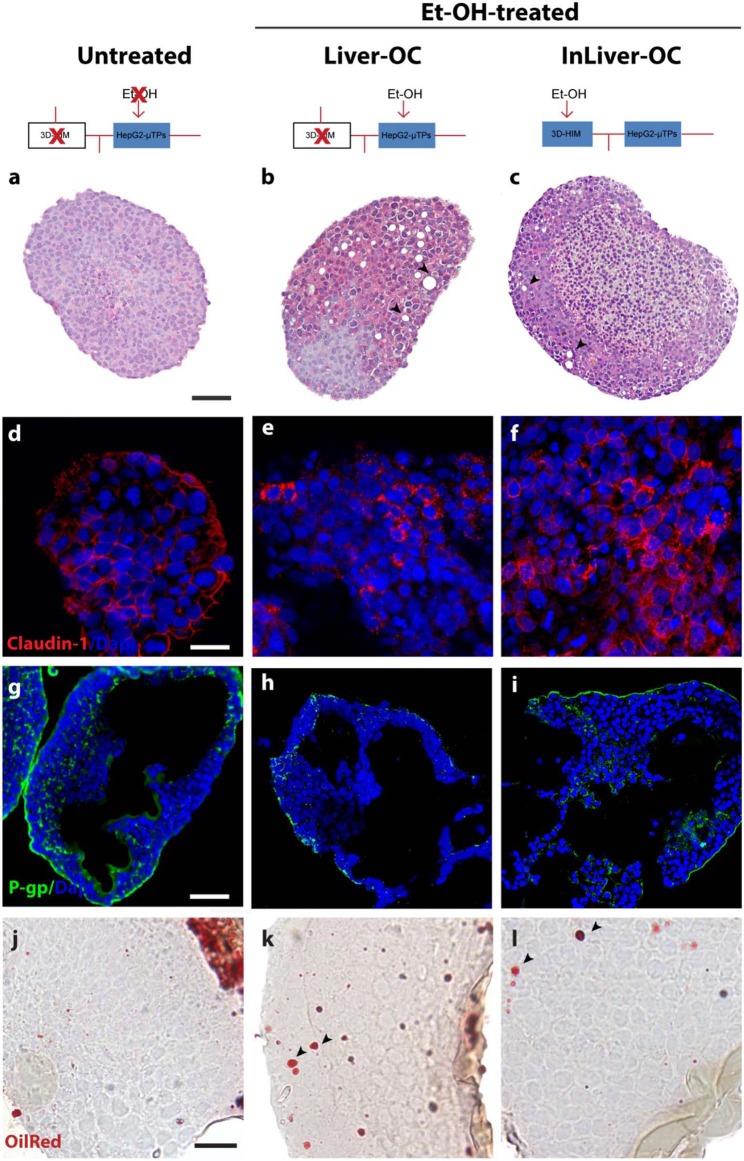
Protective role of 3D-HIM on Et-OH-induced liver cytotoxicity. Schematic representation of Et-OH administration in different configurations [untreated **(a)**, Et-OH-treated Liver-OC **(b)** and Et-OH-treated InLiver-OC**(c)**]; representative images of the histological analysis display H&E staining of untreated **(a)** Et-OH-treated Liver-OC **(b)** and Et-OH-treated InLiver-OC **(c)**; black arrowheads indicate the lipid accumulation; scale bar 100 μm; representative immunofluorescence of tight junction marker Claudin-1 of untreated **(d)**, Et-OH-treated Liver-OC **(e)** and Et-OH-treated InLiver-OC **(f)**; scale bar 50 μm; immunofluorescent staining of the protein transporter P-gp of untreated **(g)**, Et-OH-treated Liver-OC **(h)** and Et-OH-treated InLiver-OC **(i)**; scale bar 100 μm. Oil red staining reveals the lipid drop (black arrowheads) on untreated **(j)**, Et-OH-treated Liver-OC **(k)** and Et-OH-treated InLiver-OC **(l)**; scale bar 50 μm. All the experiments were performed in triplicate (*n* = 3); values represent the mean and the standard deviation.

In order to determine the intestine contribution to Et-OH metabolism, 400 mM Et-OH was administrated in three configurations of the device: Control group samples (untreated), Et-OH-treated Liver-OC (HepG2-μTPs without 3D-HIM), and Et-OH-treated InLiver-OC (device with both HepG2-μTPs and 3D-HIM). Histological characterization of untreated samples using H&E staining displayed a homogenous cell distribution around the microbeads surface of the HepG2-μTPs with the typical liver-like histotypic features, such as cuboidal hepatocyte cell shape with tight cell–cell contacts. In the Liver-OC configuration, HepG2-μTPs, although preserving the 3D epithelial organization, showed a high amount of lipid accumulation, as indicated by the black arrowheads. In contrast, in InLiver-OC configuration, HepG2-μTPs showed a small amount of lipid accumulation with a well-polarized epithelium (Figures 7a–c). Immunofluorescence staining showed that the Claudin-1 expression was higher in control groups than in Liver-OC, in which a completely damaged tight junction network was detected (Figures 7d,e, respectively). In contrast, a continuous network of tight junction was found in some area of HepG2-μTPs in InLiver-OC (Figure 7f). Moreover, in order to better characterize the liver functionality, HepG2-μTPs were stained with P-Glycoprotein (P-gp) antibody—a membrane's protein that guides the transport of the substance across the epithelium—and a qualitative analysis of the P-gp expression for untreated samples, Liver-OC, and InLiver-OC was performed, as highlighted in Figures 7g–i. Specifically, as depicted in Figure 7g, the formation of canalicular-like structures was evidenced in untreated samples. However, a slight signal of P-gp was detected in Liver-OC, which indicated no assembly of the membrane protein (Figure 7h) in this condition, while the InLiver-OC showed higher expression of this protein, well-organized both in the core and on the external surface of the microtissue (Figure 7i). Furthermore, in order to determine the lipid accumulation on HepG2-μTPs, oil red staining was assessed directly into the biochip (Figure S5) or cross-sections after withdrawing the sample from the device (Figures 7j–l). In particular, untreated samples showed a small amount of lipid accumulation. In contrast, high amount of lipid drops (stained in red, black arrowheads) were displayed in Liver-OC, and a small quantity of lipid drops was found in InLiver-OC cross-sections, as reported in Figures 7j–l. Finally, ROS were found to be over-expressed in Liver-OC compared to untreated and InLiver-OC. Figure S6 shows that the ROS expression on the Liver-OC was higher than on the InLiver-OC.

### Synergic Response of 3D-HIM and HepG2-μTPs After Et-OH Administration in InLiver-OC

**Figure 8:**
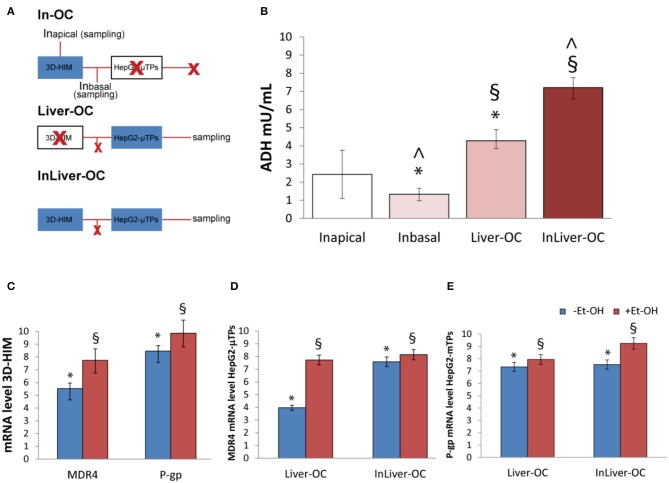
Synergic response of 3D-HIM and HepG2-μTPs after Et-OH administration. Overview of the experimental design: In-OC (device loaded with 3D-HIM without HepG2-μTPs), Liver-OC (device loaded with HepG2-μTPs without 3D-HIM) and InLiver-OC (device with both HepG2-μTPs and 3D-HIM); In_apical_ sampling indicates the tissue supernatants collected from the apical compartment of the Intestine_c_, In_basal_ indicates the tissue supernatants collected between the Intestine_c_ and Liver_c_ and sampling indicates the tissue supernatants collected downstream to the Liver_c_ **(A)**; quantification analysis of ADH activity in the Intestine_apical_ (In_apical_), in the sampling channel (In_basal_), Liver-OC, and Liver-OC; “∧” indicates that the group samples Inbasal and InLiver-OC are considered to be statistically significant (*p* < 0.001) **(B)**; quantification analysis of P-gp and MDR4 genes expression on untreated (–Et-OH) and Et-OH-treated (+Et-OH) 3D-HIM (*p < 0.0001; ^§^*p* < 0.05) **(C)**; MDR4 **(D)**; and P-gp **(E)** gene expression of untreated (–Et-OH) and Et-OH-treated (+Et-OH) HepG2-μTPs (*p < 0.001; ^§^*p* < 0.05) in the two different device configurations: Liver-OC or InLiver-OC. All the experiments were performed in triplicate (*n* = 3); values represent the mean and the standard deviation.

In order to determine the 3D-HIM as well as the HepG2-μTPs response to the Et-OH treatment in the device, three configurations were explored: In-OC (device loaded with 3D-HIM without HepG2-μTPs), Liver-OC (device loaded with HepG2-μTPs without 3D-HIM), and InLiver-OC (device with both HepG2-μTPs and 3D-HIM). In the In-OC configuration, the tissue supernatants were collected from the apical compartment of the Intestine_c_ and from the sampling channel between the Intestine_c_ and Liver_c_. In the Liver-OC configuration, the tissue supernatants were collected downstream to the Liver_c_. And, in the InLiver-OC configuration, the tissue supernatants were collected only downstream to the Liver_c_ (Figure 8A). The detection of ADH activity was used as indication of acute cell damage (Figure 8B). As deduced from the results obtained after treatment with 400 mM Et-OH, a high amount of ADH was detected in the Intestine_apical_ (In_apical_) (2.42 ± 0.65 mU/mL), indicating the enzyme activity of 3D-HIM. In addition, a slight but significant release of ADH was revealed in the sampling channel (In_basal_) (1.33 ± 0.32 mU/mL). Further, a higher amount of ADH (4.27 ± 0.42 mU/mL) was detected downstream to the Liver_c_ in the Liver-OC configuration, mimicking what occurs during acute alcoholic liver injury. At last, InLiver-OC showed the highest ADH value (7.2 ± 0.56 mU/mL) compared to the other configuration, suggesting a synergic activity of both intestinal and hepatic tissue co-cultured into the device. Meanwhile, experiments were performed to evaluate whether the Et-OH injury induced MDRs gene expression modulation. 3D-HIM as well as the HepG2-μTPs samples cultured in Liver-OC or InLiver-OC were processed to quantify the MDR gene expression by means of molecular analysis. The results indicated that MDR4 expression on 3D-HIM decreased after Et-OH injury (5.3 ± 0.6 a.u.) compared to untreated samples (7.9 ± 2.4 a.u.) (Figure 8C). Likewise, Et-OH-treated HepG2-μTPs cultured in Liver-OC displayed low values of the MDR4 gene expression (3.8 ± 0.9 a.u.) compared to untreated samples (7.2 ± 1.5). On the other hand, when HepG2-μTPs were cultured in InLiver-OC, they displayed higher values in the MDR4 gene expression (8.1 ± 1.6 a.u.) compared to Et-OH-treated Liver-OC, suggesting a protective action of intestine on hepatic function. Furthermore, an increase of P-gp gene expression (9.8 ± 2.6) was revealed on 3D-HIM after Et-OH injury, in agreement with the *in vivo* situation. P-gp gene expression slightly increased on Et-OH-treated Liver-OC (7.9 ± 1.7) but reached higher values on Et-OH treated InLiver-OC (9.2 ± 2.8) compared to untreated samples (7.5 ± 1.7) (Figures 8D,E). Lastly, the biomass production after 400 mM Et-OH treatment was measured, showing the amount of cell debrides collected in the tissue supernatants. In particular, Et-OH-treated Liver-OC showed highest biomass compared to the other conditions, indicating the major cell debrides accumulation due to the cell damage in this configuration. The biomass quantification of InLiver-OC Et-OH-treated samples correspond to the sum of the two organs contributions: 20% of Liver-OC debrides (extrapolated from the HepG2-μTPs viability assay) and In-OC debrides (Figure S7).

## Discussion

It is widely recognized that the intestinal and hepatic first-pass metabolism may depress extensively the bioavailability of drugs and xenobiotic compounds (Rowland, 1972), highlighting the urgent need for robust *in vitro* models able to recapitulate this process to support drug and nutraceutics discovery and testing. Here, an integrated microfluidic intestine–liver device (InLiver-OC) has been proposed to investigate the *in vivo* route of orally ingested Et-OH by connecting directly the two main organs (intestine and liver) involved in the first-pass metabolism. The InLiver-OC device was designed in order to allow the inter-organs crosstalk recapitulating the key features of human intestine–liver physiology and functionality. A previously established modular tissue assembling approach to fabricate the intestinal stroma (3D-ISM) composed by H-InMyoFibs embedded in their own ECM has been exploited, and then full-thickness 3D-HIM has been obtained after Caco-2 seeding and culture (De Gregorio et al., 2018a,b). Then, 3D HepG2-μTPs have been obtained by using dynamic cells seeding in a spinner flask bioreactor (Corrado et al., 2019). The microfluidic device (InLiver-OC) was designed to host the two 3D physiologically relevant *in vitro* models (3D-HIM and HepG2-μTPs) and to provide a direct link between them by connecting in a continuous fluid stream the basal side of the Intestine_c_ and the Liver_c_. As first issue, the optimization of culture media mixture to perform the organ-on-chip co-culture was faced. As reported in literature, some researchers solved the problems by using multi-organ-on-chip composed by independent modules and transferring the media from one organ module to the subsequent in a physiological sequence, which allows each organ module to be operated independently (Tsamandouras et al., 2017). However, this method leads the dilution of the culture media from one module to another, avoiding the metabolic waste accumulation, which could lead to the loss of metabolic products that are crucial for experimental purposes. In this perspective, in our InLiver-OC, the modules are directly connected and the culture medium opportunely optimized. Et-OH was selected as harmful stimulus, and the Et-OH concentration to mimic alcohol-induced liver damage was selected by performing cytotoxicity assay under different Et-OH concentration on HepG2 cells in both 2D and 3D configurations. The results indicated that Et-OH significantly increases the death rate in 2D HepG-2 monolayer samples compared to 3D HepG2-μTPs that maintained high cell vitality, suggesting that the 3D configuration was more reliable than 2D for cytotoxicity assay (Edmondson et al., 2014). In general, the main issue in reproducing the first-pass mechanism is the maintenance of a selective transport route from the apical to the basal side of the Intestine_c_. To achieve this, we fabricated a sealed chamber by using a PDMS gasket and a PMMA cylinder accommodated over the 3D-HIM. As it is well-known, once consumed, alcohol is absorbed mainly in the upper intestinal tract by diffusion and then enters the liver via the portal vein (Bishehsari et al., 2017). However, although the majority of alcohol metabolism occurs in hepatocytes, the enzymes involved in the oxidative or non-oxidative metabolism of alcohol are also present in the intestinal mucosa (Cederbaum, 2012). Mounting evidence suggests that alcohol, particularly if consumed chronically or in larger amounts, induces a deleterious effect at the intestinal district that in turn promotes inflammation through various pathways, including changes in intestinal microbiota composition and function (Mutlu et al., 2012; Engen et al., 2015), increased permeability of the intestinal mucosa (Günzel and Yu, 2013), and disruptions of the immune system of the intestinal mucosa (Bishehsari et al., 2017), as well. With this perspective, we deeply investigated the 3D-HIM response to Et-OH treatment by analyzing supernatants from apical and basal side of 3D-HIM. Surprisingly, we found that Et-OH is partly digested by 3D-HIM after 24 h, protecting HepG2-μTPs from cell mortality. In addition, in line with the literature reporting a correlation between alcohol and dysbiosis (Canesso et al., 2014), our results demonstrated the antibacterial activity on the *L. rhamnosus* strain growth of supernatants withdrawn from 3D-HIM_apical_ at T_0_ due to the presence of undigested Et-OH and of supernatants from 3D-HIM_apical_ and -_basal_ at 24 h due to the presence of Et-OH or its metabolites. Alcohol has been known for a long time to interfere with the absorption of several nutrients and to lead to small intestinal mucosal damage, thereby contributing to the increase of the intestinal trans-epithelial as well as para-cellular permeability by acting on the tight junction complex (Bode and Bode, 2003). Intestinal epithelial tight junctions' integrity is fundamental in the studies of absorption and permeation of compounds, because they form a physical barrier to the diffusion of macromolecules (Günzel and Yu, 2013). Previous studies demonstrated that the acetaldehyde, rather than Et-OH, can cause the disruption of tight junctions in Caco-2 monolayer (Atkinson and Rao, 2001). As predictable, the 3D-HIM-epithelial damage was found after Et-OH administration in InLiver-OC by analyzing the Claudin-1 signal, a transmembrane protein of the tight junctions, that form a barrier to macromolecular diffusion (Günzel and Yu, 2013). Specifically, the Et-OH-treated 3D-HIM presented a lower number of tight junctions with less organized protein structure. In addition, the TEER measurements on Et-OH-treated 3D-HIM confirmed the Et-OH deleterious effect on the epithelial barrier showing a very low TEER value compared to untreated samples. Further, the acute alcohol administration elicits basement membrane alterations determining in severe cases the separation of the epithelium from the basal lamina with the formation of sub-epithelial blisters, which ultimately cause the epithelium rupture (Singer and Brenner, 2006). In agreement, we found that in Et-OH-treated InLiver-OC, the 3D-HIM showed a basement membrane impairment reported as pixelated immunofluorescence signal of the main protein of basal lamina (Laminin V) (Haas et al., 2001). In addition, in Et-OH-treated 3D-HIM, a high quantifiable amount of mucus was produced, due to the self-defense to the high concentration of the Et-OH and its metabolites. This is in agreement with previous studies showing that chronic alcohol feeding increased mucin production in the small intestine due to alcohol-induced qualitative changes (Valatas and Kolios, 2009). However, the damage induced to the small intestine by the Et-OH involves not only the epithelial layer but also affects the stromal compartment (Casini et al., 1999). From this perspective, the availability of a 3D-HIM provided with an endogenous stroma allows to assess the complex response of the small intestine to Et-OH, otherwise not observable with the over-simplistic existing intestinal mucosa model. SHG microscopy on 3D-HIM provided high-resolution 3D images of collagen fibers in thick samples without the need for sample staining and processing and allowed us to detect the non-centrosymmetrical structure of fibrillar collagen and the textural change at stromal level resulting from Et-OH treatment (De Gregorio et al., 2019). The results indicated that Et-OH-treated 3D-HIM experienced a decrease of both the total collagen amount and of the degree of the collagen assembly, as well as a collagen stretching in the intestinal mucosa. It is reasonable to assume that ECM remodeling contributes to Et-OH intestinal mucosa injury. In terms of liver damage due to Et-OH administration we assessed the role of intestine in reducing the toxic effects by analyzing morphological and functional hepatic markers in InLiver-OC vs. Liver-OC. Our results showed a protective role of intestine able to impair the lipid accumulation and maintain quite intact the tight junction's network in Et-OH-treated HepG2-μTPs cultured in InLiver-OC. Some *in vivo* studies suggested that intestinal drug efflux transporters are involved in pharmacokinetic alterations caused by chronic alcohol exposure (Artursson and Karlsson, 1991; Sambuy et al., 2005). Here, we reported a strong increase of P-gp gene expression on 3D-HIM after Et-OH injury according to the *in vivo* situation. High value of P-gp gene expression in Et-OH-treated HepG2-μTPs in InLiver-OC indicate once again the preservation of biochemical liver function from intestine. Furthermore, in terms of protein expression, we found the correct signal of P-gp that pointed out the formation of canalicular-like structures in InLiver-OC. In addition, the high value of MDR4 gene expression indicated a physiological liver protection from toxic accumulation of bile acids and a low cytosolic ROS production in Et-OH treated HepG2-μTPs cultured in InLiver-OC samples, corroborating once again the intestine prevention to the liver injury. Even if the majority of alcohol metabolism in humans occurs in the hepatocytes cells in the liver, some enzymes involved in the oxidative metabolism of alcohol such as alcohol dehydrogenase (ADH) are also present in the intestinal mucosa. In line with the *in vivo* situation, ADH was detected in the 3D-HIM as well as HepG2-μTPs, indicating the enzyme activity of InLiver-OC after Et-OH treatment. Liver-OC also showed high amount of ADH indicating acute alcoholic liver injury. These data confirmed the synergic contribution of both tissues when co-cultured into InLiver-OC together. Lastly, it should also be noted that the biomass production after Et-OH treatment indicated the comparable debris accumulation when single tissues culture (In-OC or Liver-OC) or co-culture (InLiver-OC) were performed in the device. These results suggested that the culture medium guaranteed the physiological function of both intestine and hepatic equivalent tissues and did not induce hepatic damage due to intestinal metabolic waste accumulation in InLiver-OC. Taken together, these results point out the stromal and epithelial damage of the 3D-HIM under orally administrated xenobiotic and the protective intestinal role in attenuating the Et-OH-induced liver cytotoxicity.

## Conclusion

In conclusion, in this study, we developed a microfluidic InLiver-OC with the aim of reproducing the first pass metabolism. We have observed the intestine–liver crosstalk in the metabolic and absorptive properties. By using the Et-OH as a harmful stimulus, we have shown that this device can be used as a tool to reproduce the first-pass metabolism of drugs and xenobiotics. Our proposed model is a more predictive platform than the 2D cell culture, resembling the physiological way route of the *in vivo* first-pass metabolism. It is also suitable to study the interaction of the gastrointestinal tract, the effects of nutraceutical substances, and the uptake of compounds. InLiver-OC can be adapted to co-culture human gut microbiome to provide a versatile platform to investigate host-microbiome interaction in a multi-organs platform. Finally, this microdevice is expected to reduce the number of drug candidates and accelerate the pre-clinical screening process reducing animal testing.

## Data Availability Statement

The datasets generated for this study are available on request to the corresponding author.

## Author Contributions

GI and PN conceived the idea and critically revised the manuscript with input from the entire team. VD, GI, and FU worked on the study conception and design, analyzed and interpreted the data, and drafted the manuscript. VD and MT carried out biological experiments. VR performed electrophysiological measurements. BC designed and microfabricated the device. FU performed fluidodynamic simulations experiments. All authors have read and approved the final draft.

### Conflict of Interest

The authors declare that the research was conducted in the absence of any commercial or financial relationships that could be construed as a potential conflict of interest.
