# Supplementary material for: Intestine-Liver Axis On-Chip Reveals the Intestinal Protective Role on Hepatic Damage by Emulating Ethanol First-Pass Metabolism
Source: Front Bioeng Biotechnol. 2020 Mar 17;8:163. doi: 10.3389/fbioe.2020.00163 (PMC7090126; doi:10.3389/fbioe.2020.00163)
Supplement: Supplementary file 1 [file Data_Sheet_1.pdf]

Supplementary Material

Materials and methods:

Figure. S1

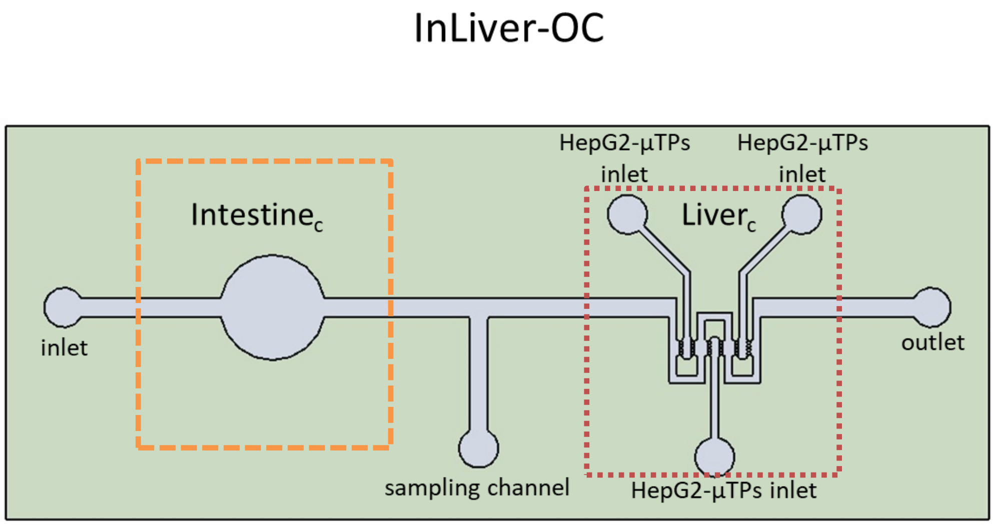

Figure. S1 Schematic representation of the InLiver-OC

Top view of the InLiver biochip with Intestine compartment (Intestine$_c$, orange dotted line) and Liver compartment (Liver$_c$, red dotted line); culture medium inlet was indicated as inlet; the HepG2-µTPs inlet showed the HepG2-µTPs loading points; sampling channel and outlet were showed as the collection site between the Intestine$_c$ and Liver$_c$ and downstream to the outlet Liver$_c$, respectively.

Figure. S2 Circuit for TEER measurement

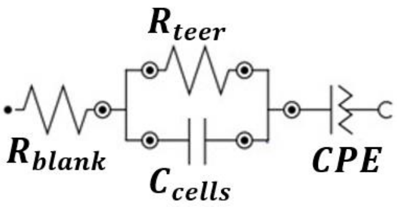

Figure. S2 Simplified equivalent circuit used to fit and analyze the impedance spectrum of cellular systems. R_teer includes resistive contribution of both the cell membrane (R_cells) and tight junction (R_tight). However, since these equivalents elements are in parallel, and since R_cells >> R_(tight), R_teer= R_tight // R_cells ≈ R_tight.

Results

Figure. S3 Growth curve

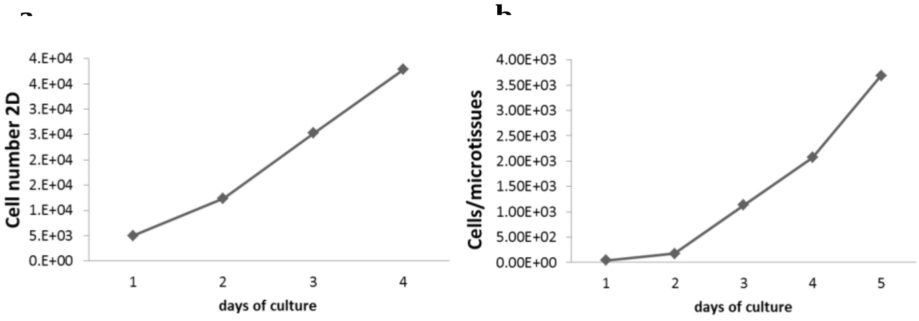

Figure. S3 The graphs indicated the cell number growth during the culture time in 2D (a) and 3D configurations (b).

Figure. S4 Regression line of Et-OH Dose-response

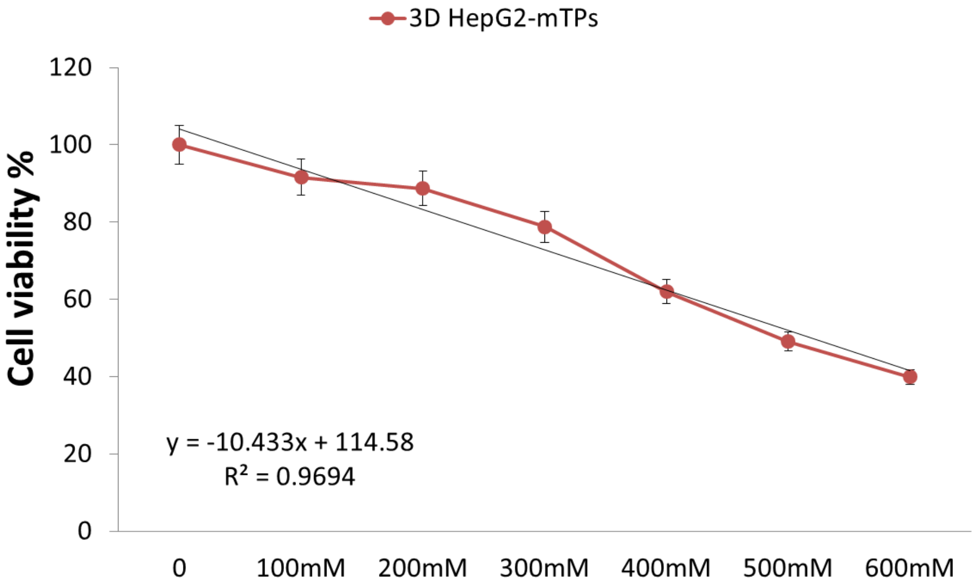

Figure. S4 The graph indicates the linear regression line of the Et-OH dose-response curve

Figure. S5 Oil red staining on chip

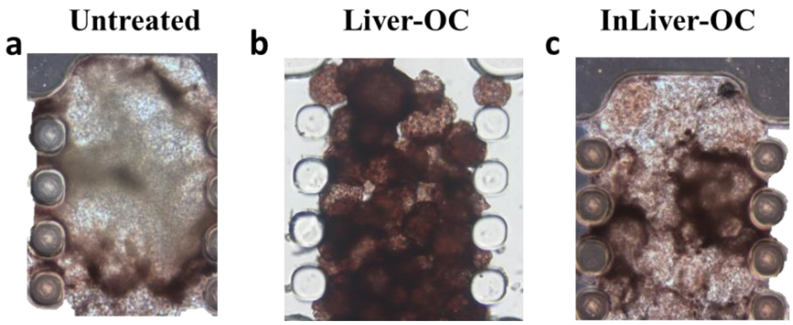

Figure. S5 Oil red staining was performed directly into the microfluidic device in order to evaluate the lipid accumulation in different configurations: untreated (a), Liver-OC (b), InLiver-OC (c).

Figure. S6 ROS expression

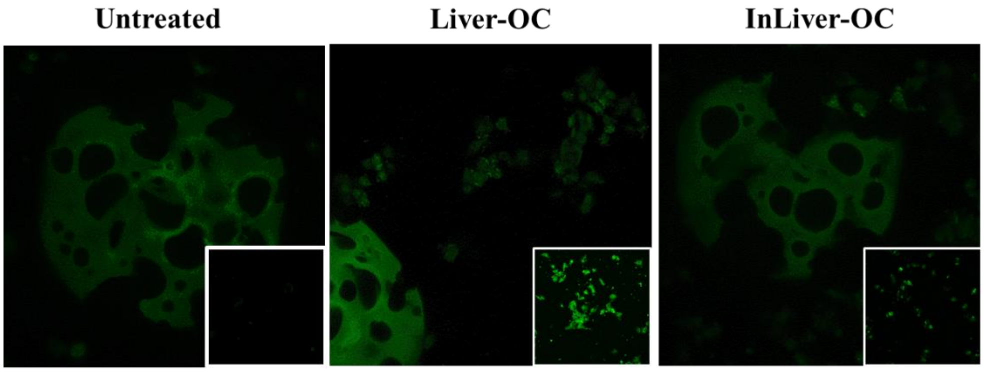

Figure. S6 Representative immunofluorescence images and high magnification insets of the HepG2-μTPs highlight a high production of ROS stained in green in Liver-OC (b) compared to InLiver-OC (c). Untreated samples revealed no signal for ROS (a). Scale bar 75 μm.

Figure. S7 Biomass quantification

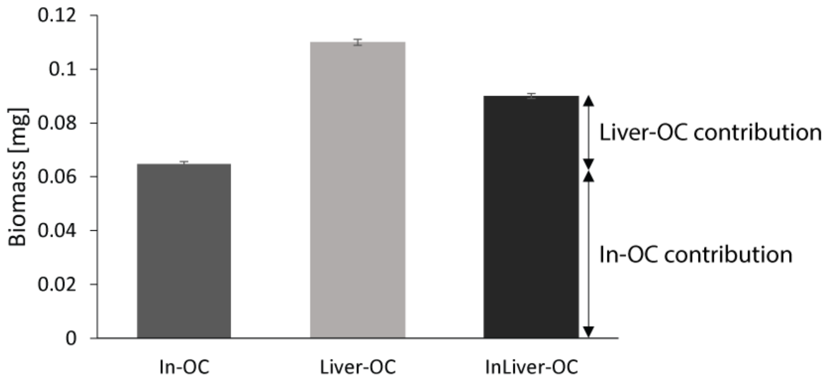

Figure. S7 The graph indicated the biomass quantification of In-OC, Liver-OC and InLiver-OC_supernatants after Et-Oh treatment.
